# Effect of global warming on the potential distribution of a holoparasitic plant (*Phelypaea tournefortii*): both climate and host distribution matter

**DOI:** 10.1038/s41598-023-37897-1

**Published:** 2023-07-03

**Authors:** Renata Piwowarczyk, Marta Kolanowska

**Affiliations:** 1 Center for Research and Conservation of Biodiversity, Department of Environmental Biology, Institute of Biology, Jan Kochanowski University, Uniwersytecka 7 Street, 25-406 Kielce, Poland; 2 Faculty of Biology and Environmental Protection, Department of Geobotany and Plant Ecology, University of Lodz, Banacha 12/16, 90-237 Lodz, Poland

**Keywords:** Climate-change ecology, Ecological modelling

## Abstract

*Phelypaea tournefortii* (Orobanchaceae) primarily occurs in the Caucasus (Armenia, Azerbaijan, Georgia, and N Iran) and Turkey. This perennial, holoparasitic herb is achlorophyllous and possesses one of the most intense red flowers among all plants worldwide. It occurs as a parasite on the roots of several *Tanacetum* (Asteraceae) species and prefers steppe and semi-arid habitats. Climate change may affect holoparasites both directly through effects on their physiology and indirectly as a consequence of its effects on their host plants and habitats. In this study, we used the ecological niche modeling approach to estimate the possible effects of climate change on *P. tournefortii* and to evaluate the effect of its parasitic relationships with two preferred host species on the chances of survival of this species under global warming. We used four climate change scenarios (SSP1-2.6, SSP2-4.5, SSP3-7.0, SSP5-8.5) and three different simulations (CNRM, GISS-E2, INM). We modeled the species’ current and future distribution using the maximum entropy method implemented in MaxEnt using seven bioclimatic variables and species occurrence records (*Phelypaea tournefortii* – 63 records, *Tanacetum argyrophyllum* – 40, *Tanacetum chiliophyllum* – 21). According to our analyses, *P. tournefortii* will likely contract its geographical range remarkably. In response to global warming, the coverage of the species’ suitable niches will decrease by at least 34%, especially in central and southern Armenia, Nakhchivan in Azerbaijan, northern Iran, and NE Turkey. In the worst-case scenario, the species will go completely extinct. Additionally, the studied plant's hosts will lose at least 36% of currently suitable niches boosting the range contraction of *P. tournefortii*. The GISS-E2 scenario will be least damaging, while the CNRM will be most damaging to climate change for studied species. Our study shows the importance of including ecological data in niche models to obtain more reliable predictions of the future distribution of parasitic plants.

## Introduction

The climate is one of the key factors influencing the distribution of plants^1,2^, and global warming has become a major threat to global biodiversity^3–8^ and ecosystem balance^9^. The current extinction rates are around 1000 times higher than the background rates^10^. Temperature and precipitation pattern alterations also result in species redistribution, which will accelerate^11^. Species occurrence information is essential to conservation and management plans, but these data are often incomplete. At the beginning of the XXI century, the increasing availability of geospatial data led to a rapid improvement of analytical methods. Scientists developed numerous predictions of Earth’s future climate to evaluate the impact of temperature and precipitation alteration on living organisms. Global climate models or general circulation models (GCMs)—based on the general principles of dynamics and thermodynamics describing the atmosphere and ocean dynamics—became a base for numerical analyses^12,13^of climate changes during the past, present, and future^14^. Ecological niche modeling (ENMs) provides a tool for mapping species distribution and can produce reliable and repeatable data useful in biodiversity and conservation management^9,15^. For example, according to the most recent summary, the impact of global warming was evaluated for less than 2% of plant species^16^.

Parasitic plants account for 1.6% of all angiosperms, with 4750 species representing 26 families^17^. These plants occur in all terrestrial vegetation types; some are widespread agricultural pests^18^. These parasites developed an intrusive organ called a haustorium, a vascular bridge that connects the parasite with the host’s vascular tissue. Approximately 10% of described parasitic species cannot photosynthesize (holoparasitic), so they harbor carbohydrates direct from the phloem; the remaining harbors just water and nutrients from the host xylem (hemiparasitic). About 40% of plant parasites are aerial, and 60% are root parasites. Parasites allogenic engineers or facilitators because they affect the growth, reproduction, and allometry of other species and ecosystems due to their effects on trophic interactions with other organisms^19–21^. Parasitic plants may also cause a cascade effect in an ecosystem by altering interspecies relationships at various trophic levels^22^. So, they are keystone species to ecosystems.

Without a doubt, global warming affects the distribution of parasitic plants and their hosts by altering the temperature and precipitation and changing the local abiotic conditions. However, the overlap of suitable areas in the future between parasitic plants and their hosts is practically unknown. The occurrence of parasites, which are highly specialized species, is determined by climate and also by their hosts^11,23^. Consequently, holoparasites in natural and seminatural habitats are often rare and endangered^24,25^. Although published data are limited^26–33^, ecological niche modeling (ENM) techniques can help evaluate global warming impacts on heterotrophic plants, especially holoparasites. Niche modeling makes it possible to forecast the potential future distribution of various species^34,35^, providing helpful information for environmental monitoring, conservation, and management of parasitic plants and their hosts.

Orobanchaceae occurs worldwide, and it is the largest parasitic plant family with 102 genera and over 2100 species^17^. Possessing various trophic modes, it is a valuable model for studying parasitism evolution and physiology^36^. The tribe Orobancheae is the oldest and most species-rich of holoparasites, with a crown age dating to the mid-Miocene^37,38^. The Mediterranean Basin and the Caucasus region of western Asia are centers of extant diversity for the most diverse genera in the Orobancheae^25^ and one of the world’s biodiversity hotspots^39^.

The genus *Phelypaea* L. (≡ *Diphelypaea* Nicolson, *nom. illeg*.) (Orobanchaceae) includes three species: *P. coccinea* (M. Bieb.) Poir., *P. boissieri* (Reut.) Stapf, and *P. tournefortii* Desf. These species occur primarily in the Caucasus and less frequently in the Middle East, Crimea, and Balkans. These perennial, holoparasitic herbs are achlorophyllous and produce one of the most intense red flowers among all plants worldwide. They occur as parasites on the roots of *Tanacetum*, *Centaurea*, *Psephellus*, rarely *Klasea*, and *Cousinia* (all Asteraceae genera).

**Figure 1:**
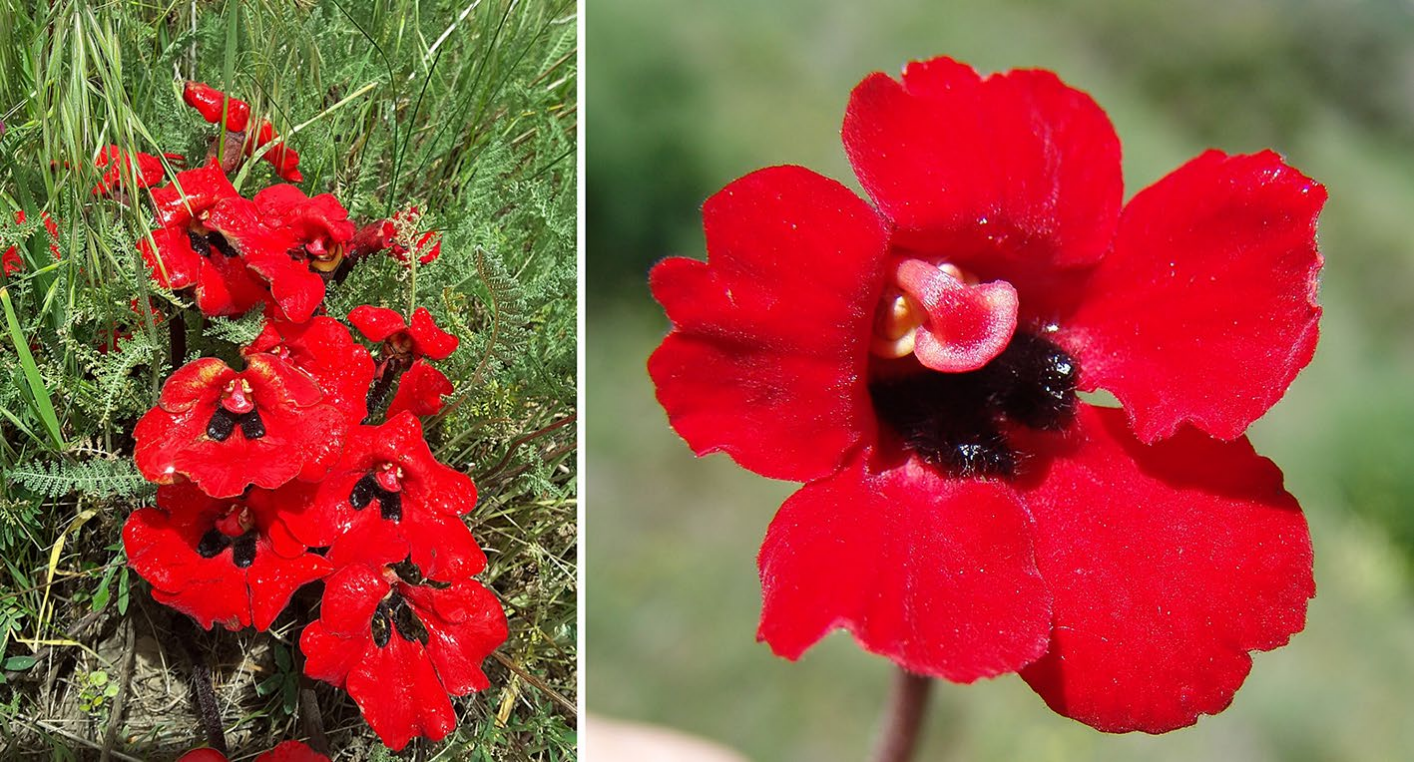
General habit (left) and closeup of a flower (right) of *Phelypaea tournefortii*. Photos: R. Piwowarczyk.

For this study, we selected *P. tournefortii* (Fig. 1), occurring primarily in the Caucasus (Armenia, Azerbaijan, Georgia, and N Iran) and Turkey. It parasitizes on roots of *Tanacetum*, mainly *T*. *polycephalum* subsp. *argyrophyllum* (K. Koch) Podlech [syn. *T. argyrophyllum* (C. Koch) Tzvel.], *T. chilophyllum* (Fisch. & Mey.), and *T. canescens* DC. [*Pyrethrum canescens* (DC.) Boiss.]^24^. Other previously reported *Anthemis* L., *Pyrethrum myriophyllum* (Willd.) C.A.M. [*Tanacetum polycephalum* subsp. *duderanum* (Boiss.) Podlech]^40^, which were not confirmed to be hosts of *P. tournefortii*. The species studied generally can be found in dry steppe and semi-arid habitats. In Armenia, which is especially rich in *P. tournefortii* populations, it usually grows at (1200)1800–2100(2500) m above sea level. The species flower from May to June; its population bears a few dozen to rarely a few hundred shoots^24^. The intense red-colored flowers (Fig. 1) of this species are rich in anthocyanins, and this pigment has not been reported previously in such an unprecedentedly large quantity in any plant^41^.

In this study, we used the ecological niche modeling (ENM) approach to estimate the possible effects of climate change on the distribution of *P. tournefortii*. Also, to evaluate the survival chances of this parasitic plant considering its relationships with two of the most commonly observed and preferred host species. Moreover, we aim to identify the key climatic factors affecting the distribution of studied species. In this context, we tested two hypotheses: (1) climate change directly affects the distribution of *P. tournefortii*, and (2) the distribution of *P. tournefortii* hosts is also impacted, indirectly affecting the parasite occurrence.

## Methods

### Species occurrence data

The database *Phelypaea tournefortii* records were compiled based on examining specimens of 12 herbaria (BAK, BP, ERE, ERCB, G, LE, KTC, MW, NY, P, W, WU; acronyms follow *Index Herbariorum*^42^); extensive fieldwork carried out from May to August in Armenia and Georgia from 2014 to 2022, during seven expeditions to all provinces; and revised photographs from Plantarium (https://www.plantarium.ru/lang/en.html) and iNaturalist (https://www.inaturalist.org/) databases. We identified host plants in the field by removing the soil with a gardening shovel to the level of attachment of the haustoria to the host roots or by analyzing herbarium photographs containing an attached host. In the field, we analyzed ca. 30 soil pits with attached hosts. Species were collected and identified by Renata Piwowarczyk. Vouchers were deposited in the Herbarium of Jan Kochanowski University in Kielce (KTC), Poland. The collection is not numbered, but the herbarium sheets are stored in a separate section under the name “Caucasus”, and the species are sorted alphabetically, in boxes labeled with the species name. Field studies and plant collection complied with relevant local, institutional, national, and international guidelines, permissions, or legislation, and we obtained the necessary permissions. We did not collect germplasm for the study.

**Figure 2:**
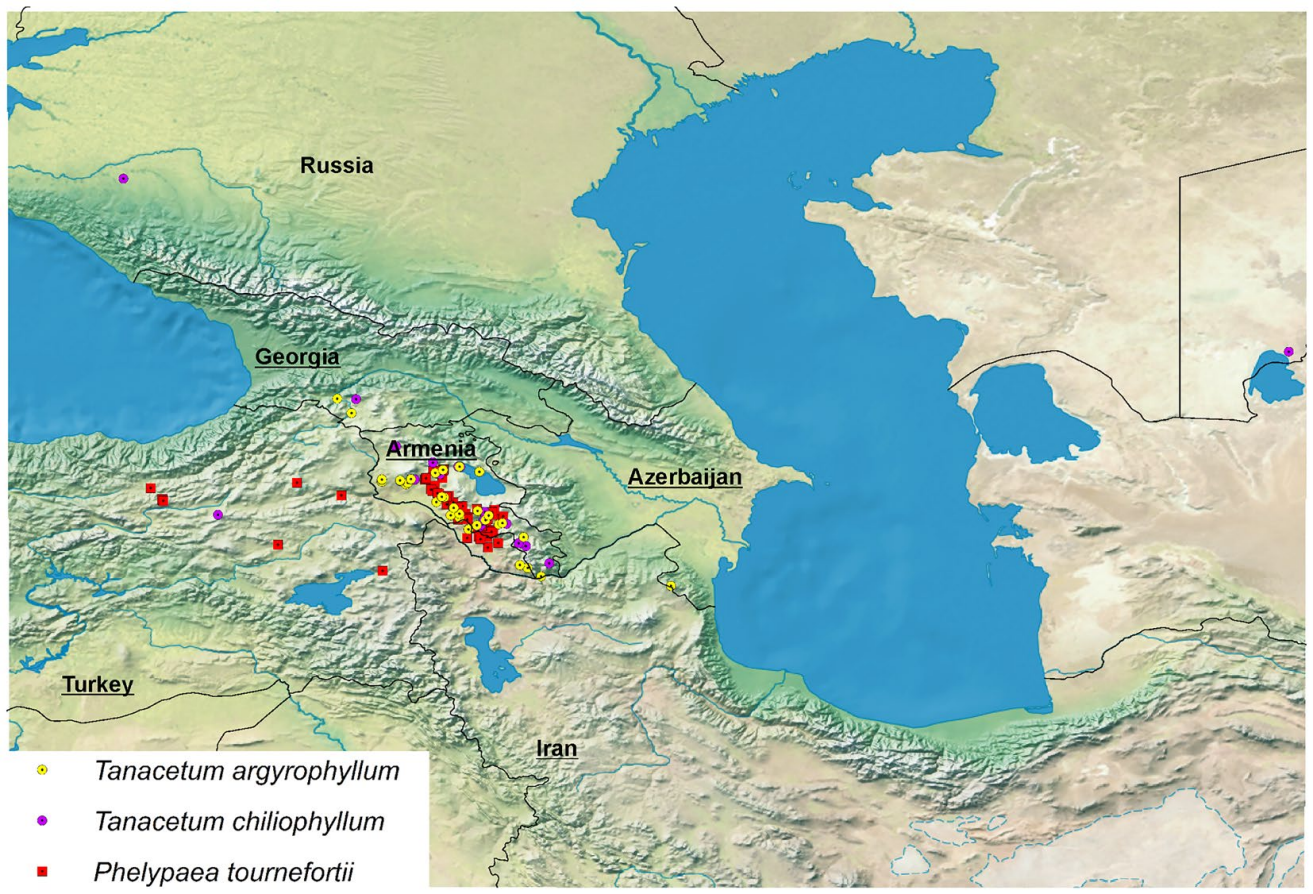
Locations of species studied used in analyses. Map generated in ArcGIS, country names added using CorelDraw 2020.

We downloaded records on the parasite hosts from GBIF (Global Biodiversity Information Facility^43,44^). The correctness of species identification was verified using photos and scans of herbarium specimens listed in GBIF. We only used records that we could georeferenced with the precision of 1 km. We employed spatial thinning to reduce sampling bias, using SDMtoolbox 2.3 for ArcGIS^45,46^. The location data were spatially filtered at 1 km^2^ to maximize the number of spatially independent localities. The final database included 63 records for *Phelypaea tournefortii,* 40 for *Tanacetum argyrophyllum*, and 21 for *Tanacetum chiliophyllum* (Annex 1, Fig. 2).

### Niche modelling and analysis

We modeled the species’ current and future distribution using the maximum entropy method implemented in MaxEnt version 3.3.2^34,47,48^, a presence-only approach. This method is currently the most popular species modeling technique^49^ and it is based on the search for patterns in the distribution of environmental factors at points with confirmed species occurrence^50^. MaxEnt is efficient in modeling rare species, even with small sample sizes^51^. To maximize the reliability of the models, we followed suggestions presented by Lissovsky and Dudov^50^ – we eliminated aggregations of occurrence points and minimized correlations of environmental predictors.

We obtain the bioclimatic variables from WorldClim v. 2.1 ^52^(Annex 2). The variables were in 30 arc-seconds (~ 1 km). While some studies indicated that MaxEnt is robust to predictor collinearity and exclusion of highly correlated variables has little impact on the model^53^, of 19 available variables, 12 were removed due to auto-correlation (> 0.7) as indicated by Pearson’s correlation coefficient computed using SDMtoolbox 2.3 for ArcGIS^45,46^ (Annex 3). We used to model the following bioclimatic variables: BIO1 (annual mean temperature), BIO2 (mean diurnal range [mean of monthly (max temp—min temp)]), BIO4 (temperature seasonality (standard deviation × 100)); BIO8 (mean temperature of wettest quarter), BIO9 (mean temperature of driest quarter), BIO12 (annual precipitation), and BIO15 (precipitation seasonality (coefficient of variation). We restricted the area of the analysis to 34.10–447.54°N and 38.05°–57.9°E^54^.

Predictions of the potential future distribution of *Phelypaea tournefortii* in 2080–2100 were made using four projections of four Shared Socio-economic Pathways (SSPs): 1–2.6, 2–4.5, 3–7.0, and 5–8.5^55–57^. SSPs are trajectories representing various future socio-economic and political environments together with the expected level of radiative forcing in 2100 (up to 8.5 W/m^2^)^58–60^. We used three of the simulations of future climate developed by Coupled Model Intercomparison Project Phase 6 (CNRM), Goddard Institute for Space Studies (GISS), and Institute for Numerical Mathematics (INM). We chose projections due to the differences between simulated maximum temperature and precipitation within the study area (Annex 4). We also downloaded all variables describing future climate from WorldClim v. 2.1 (www.worldclim.org/data/cmip6).

Unfortunately, soil characters cannot be included in any modeling of future changes in the coverage of suitable areas^61,62^ due to the lack of future models of soil types distribution. Some researchers use the soil to predict future distribution, assuming this variable will not change in the next 100 years. We considered this cautiously^63^ since soil properties result from the local substrate, climate, and vegetation and greatly depend on the composition of local microbiota^64^. Numerous studies showed that climate will also affect these organisms^65,66^. Thus, predicting future soil characteristics changes is extremely difficult. For these reasons, we chose not to include soil as a variable.

Because previous studies indicated that MaxEnt’s default number of iterations (500) is acceptable only for simple modes^50^, we set the maximum number of iterations to 10,000 and the convergence threshold to 0.00001. The neutral (= 1) regularization multiplier value and auto features were used. All samples were added to the background. The “random seed" option provided a random test partition and background subset for each run, and 20% of the samples were used as test points. The run was performed as a bootstrap with 100 replicates. We set the output to logistic. In addition, the “fade by clamping” function in MaxEnt precluded extrapolations outside the environmental range of the training data^67^. We used ArcGIS 10.6 (Esri, Redlands, CA, USA) for all analyses of GIS data. We evaluated models using the area under the curve (AUC)^68,69^ and True Skill Statistic (TSS)^70,71^.

We created Several sets of models. First, the future distribution of *P. tournefortii* was estimated using exclusively bioclimatic data (bioclims). Then, the potential distribution of the plants’ hosts was estimated for all analyzed climate change scenarios. Finally, the hosts’ models were included as separate variables in a second set of analyses for *P. tournefortii* (bioclims + hosts).

We used SDMtoolbox 2.3 for ArcGIS^45,46^ to visualize changes in the distribution of suitable areas of the parasite and its hosts due to global warming. Because MaxEnt does not use absence data, numerous authors criticized this ability to predict threshold values^35,72^.The calculated maximum Kappa value (Table 1) was used as a presence threshold in our analyses. All models were converted into binary rasters based on maximum Kappa value—using the Goode homolosine as a projection—to compare the model for current climatic conditions with future predictions. We created predicted niche occupation (PNO) profiles—which integrate species suitability (models obtained with MaxEnt) with a single climatic variable—to visualize the environmental preferences of the parasite studied^73^. We also calculated overall change by comparing the current potential coverage of suitable niches (area of stable range + area suitable in the present time but not suitable in the future) with the future coverage (area of stable range + calculated range expansion for the future).

**Table 1:** Results of model evaluation tests.

Species	AUC (standard deviation)	TSS	Max kappa
*Phelypaea tournefortii*	0.992 (0.001)	0.92	0.47
*Tanacetum argyrophyllum*	0.994 (0.001)	0.94	0.48
*Tanacetum chiliophyllum*	0.977 (0.015)	0.90	0.44

## Results

### Model evaluation and limiting factors

The scores of the values of both, TSS and AUC tests were high with 0.90–0.94 and 0.965–0.990, respectively (Table 1), which indicates the high accuracy of the models.

**Figure 3:**
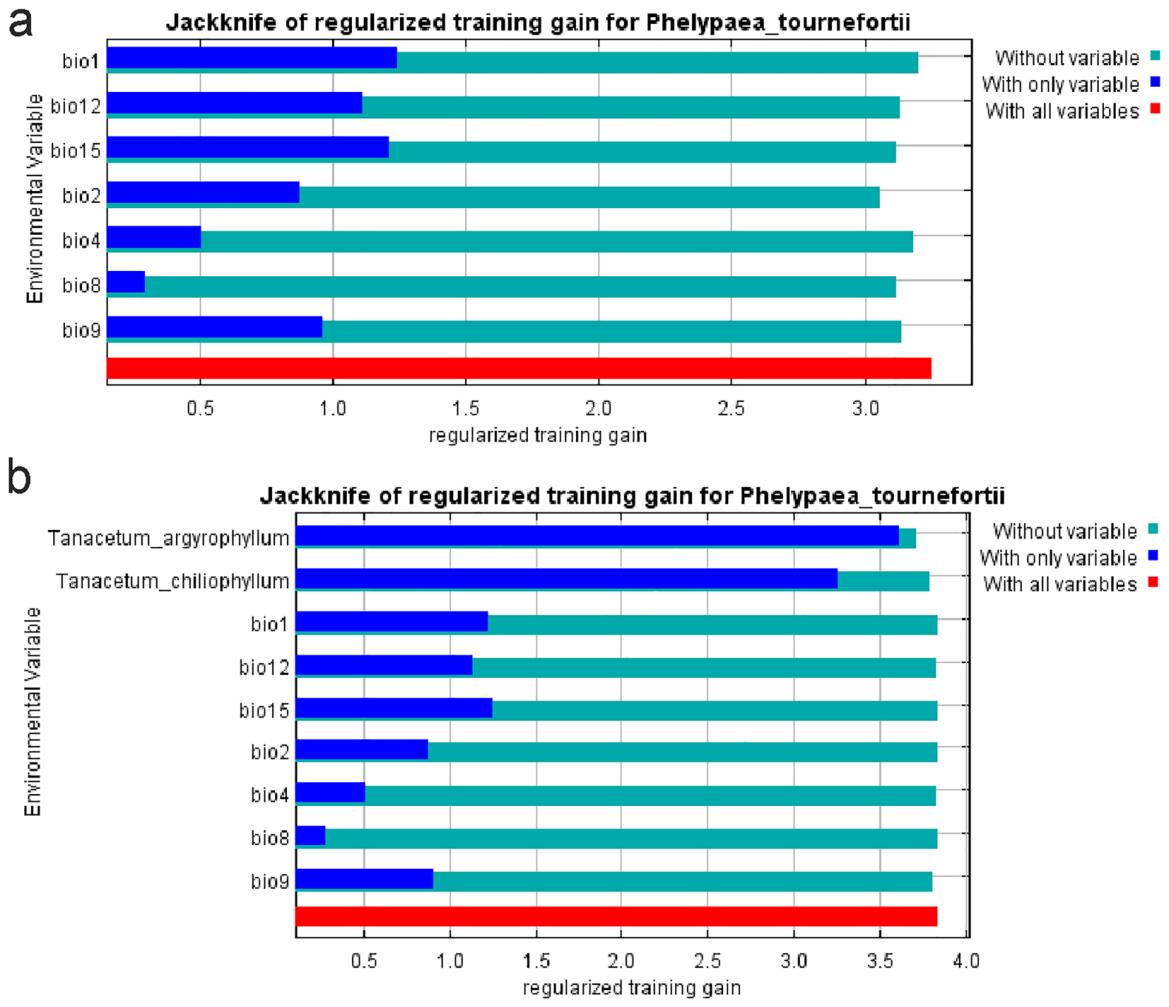
Results of the jackknife test of variable importance. (**a**) models of *Phelypaea tournefortii* based exclusively on bioclimatic variables, (**b**) models of *P. tournefortii* based on host distribution models and bioclimatic variables. Graphs generated in MaxEnt.

According to the jackknife test, the most representative in the models based exclusively on bioclimatic data were BIO1 (annual mean temperature) (Fig. 3), BIO2 (mean diurnal range), and BIO15 (precipitation seasonality). However, the crucial factors limiting the distribution of the studies species in combined models (bioclims + hosts) were distributions of *T. argyrophyllum* and *T. chiliophyllum* (Fig. 3)*.* We provide the results of the jackknife test for *P. tournefortii* hosts in Annex 5.

**Figure 4:**
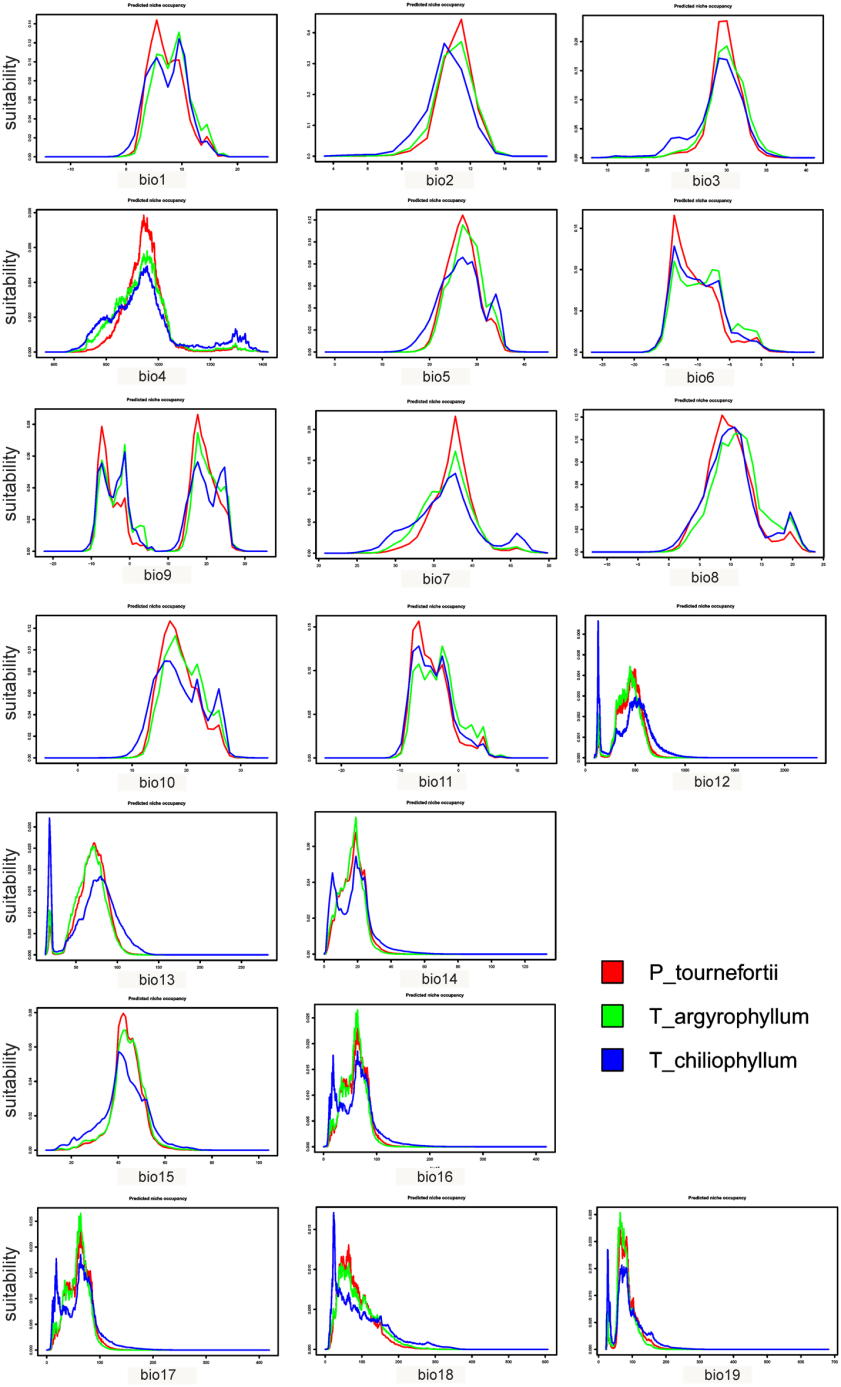
Predicted niche occupancy profiles created for *Phelypaea tournefortii* and its hosts. Graphs generated in RStudio.

*P. tournefortii* occurs in areas characterized by relatively low annual mean temperature (3–10 °C) and medium mean diurnal range of 10–13 °C (Fig. 4). The preferred isothermality ranges from 2.7 to 3.3 and temperature seasonality from 9 to 10.5 (Fig. 4). Considering the extrema of temperatures, the favored max temperature of warmest month is 22–33 °C (Fig. 4) and min temperature of coldest month − 15 to − 5 °C (Fig. 4). The annual precipitation in the areas where studied species occurs ranges between 150 and 350–750 mm (Fig. 4). The calculated precipitation seasonality in the suitable areas was 35–55 (Fig. 4). *P. tournefortii* is resistant to low precipitation as precipitation of the driest month is about 0–35 mm and precipitation of the driest quarter is 10–110 mm (Fig. 4).

### Potential future distribution of *Phelypaea tournefortii*

**Figure 5:**
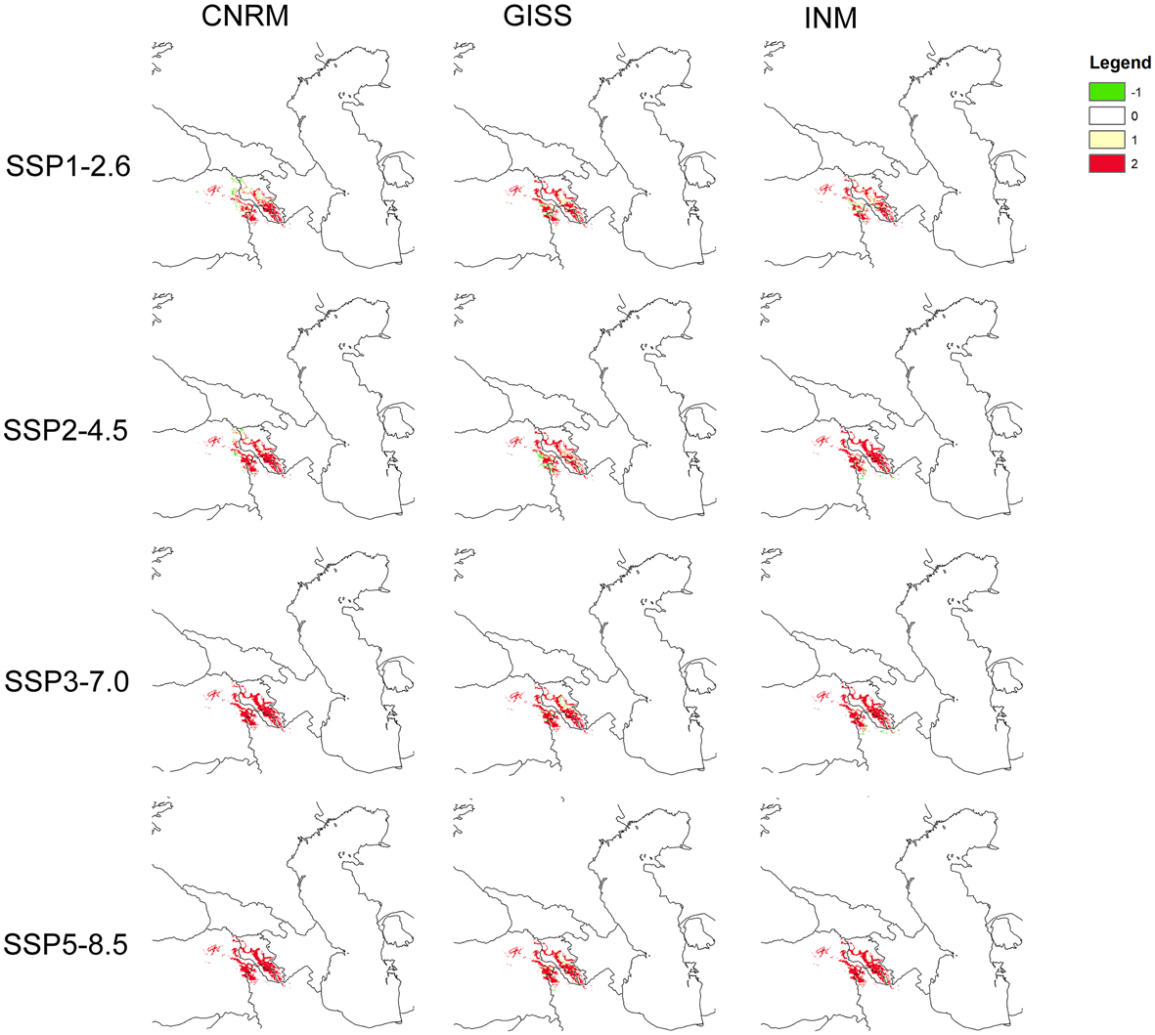
Predicted future distribution of suitable areas for *Phelypaea tournefortii* based on climate data only according to various simulations: CNRM, GISS, INM. Legend: range expansion (-1, green), absent (0, white), present in both present time and future models (1, light yellow), range contraction (2, red). Maps generated in ArcGIS.

**Figure 6:**
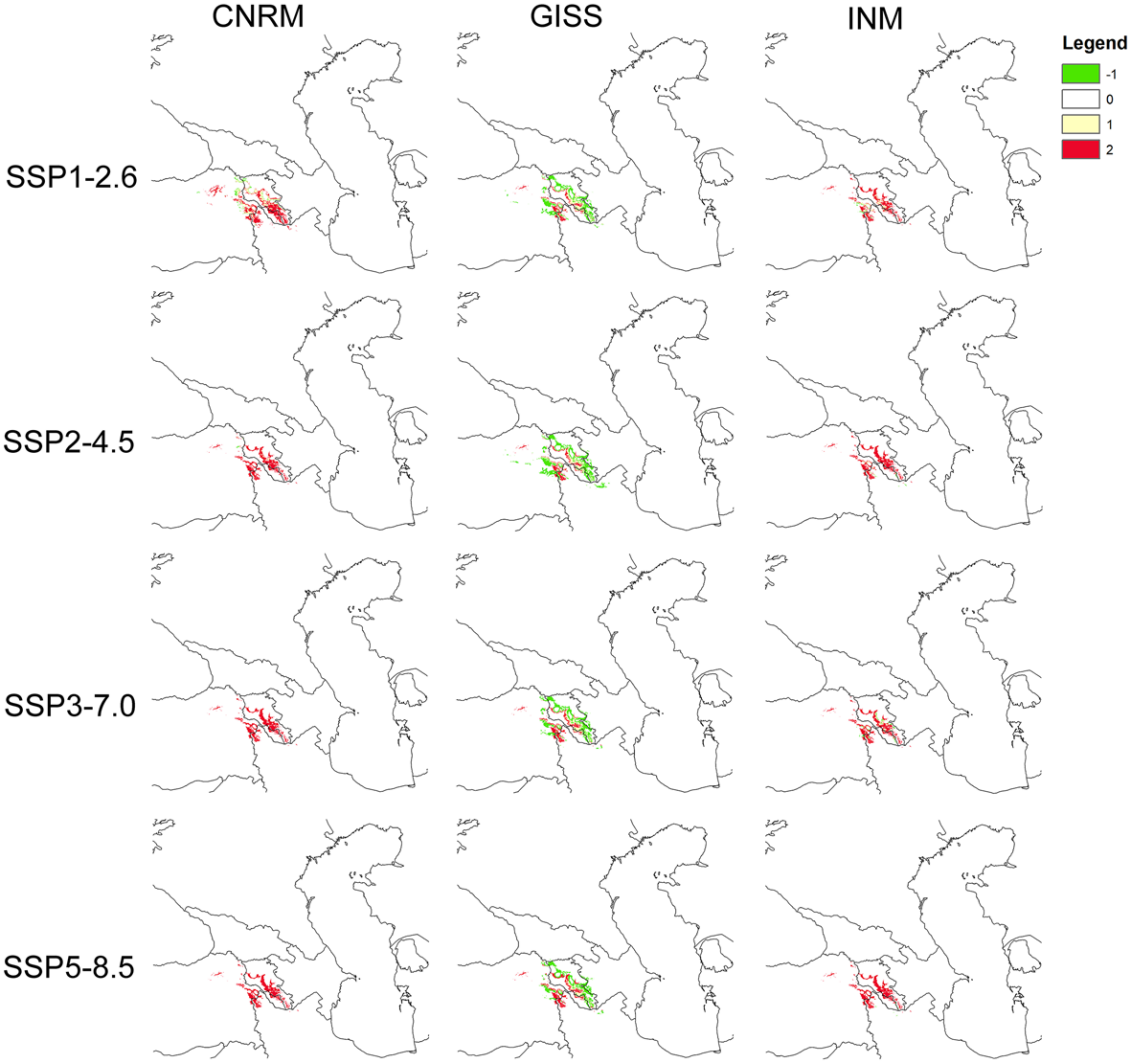
Predicted future distribution of suitable areas for *Phelypaea tournefortii* based on climate data and host models according to various simulations: CNRM, GISS, INM. Legend: range expansion (-1, green), absent (0, white), present in present and future models (1, light yellow), range contraction (2, red). Maps generated in ArcGIS.

The models indicated that *Phelypaea tournefortii* and its hosts would lose significant suitable areas due to global warming (Figs. 5, 6; Table 2). GISS-E2 presented the most favorable scenario for studied species, while CNRM simulated the most damaging climate change. Generally, the future range contraction of *P. tournefortii* is higher in models based exclusively on climatic data. Including the plants’ hosts in the analyses reduced the expansion of available areas due to the habitat loss predicted for studied hosts in almost all CNRM models (SSP2-4.5, SSP3-7.0, SSP5-8.5) and two INM models (SSP2-4.5, SSP5-8.5). On the other hand, predicted future host presence was beneficial for future occurrence of *P. tournefortii* in GISS-E2 projections. We provide the changes in the coverage of suitable areas for *P. tournefortii* hosts in Annex 6.

**Table 2:** Changes in the coverage (km^2^) of suitable areas for *Phelypaea tournefortii* estimated by the models using only bioclimatic variables (bioclims) and using the host’s models as variables (bioclims + host).

SSPs	Scenario	Range expansion		Range contraction		Overall change	
		*Phelypaea tournefortii* (bioclims only)	*Phelypaea tournefortii* (bioclims + hosts)	*Phelypaea tournefortii* (bioclims only)	*Phelypaea tournefortii* (bioclims + hosts)	*Phelypaea tournefortii* (bioclims only)	*Phelypaea tournefortii* (bioclims + hosts)
CNRM	SSP1-2.6	343.171	343.171	6.713.889	6.713.889	− 70%	− 70%
	SSP2-4.5	682.604	77.756	14.244.211	8.601.703	− 82%	− 94%
	SSP3-7.0	44.859	0.000	16.384.730	9.038.330	− 99%	− 100%
	SSP5-8.5	0.000	0.000	16.557.437	9.039.825	− 100%	− 100%
GISS-E2	SSP1-2.6	702.790	9.385.239	12.124.626	3.716.564	− 69%	63%
	SSP2-4.5	1.359.226	11.803.884	11.368.005	3.700.115	− 60%	90%
	SSP3-7.0	719.986	9.639.440	14.427.385	4.817.103	− 83%	53%
	SSP5-8.5	741.668	8.843.194	14.855.040	5.801.009	− 85%	34%
INM	SSP1-2.6	288.593	349.152	10.572.506	6.814.074	− 62%	− 72%
	SSP2-4.5	599.615	150.277	15.198.211	8.502.266	− 88%	− 92%
	SSP3-7.0	595.129	674.379	15.512.971	7.812.933	− 90%	− 79%
	SSP5-8.5	299.060	113.643	15.982.495	9.026.368	− 95%	− 99%

The future models were obtained using made using four projections of four shared socio-economic pathways (SSPs) and 12 scenarios.

Predicted future suitable areas for *P. tournefortii* based on climate data only according to various simulations (Fig. 5) indicate overall potential range contraction in all scenarios, especially in central and southern Armenia, Nakhchivan in Azerbaijan, northern Iran and, northeastern Turkey. The most damaging scenarios are SSP3-7.0 and SSP5-8.5. The predicted future suitable areas for *P. tournefortii* based on climate data and host models according to various simulations (Fig. 6) look similar, especially for the CNRM (Fig. 6) and INM (Fig. 6) projections. However, when we include the hosts' distribution models, these showing lower contractions values in numerous predictions. GISS projection showed different results (Fig. 6) with a significant range expansion predicted in Armenia, Nakhchivan in Azerbaijan, eastern Turkey, and northern Iran, and slightly in western Azerbaijan.

## Discussion

Based on conducted analyses, global warming will cause a significant loss of suitable areas for *Phelypaea tournefortii* and its hosts, especially in central and southern Armenia, Nakhchivan in Azerbaijan, northern Iran, and northeastern Turkey (Figs. 5, 6; Table 2). GISS-E2 was the most favorable for studied species, while CNRM was the most damaging. *Phelypaea tournefortii* occurs in areas characterized by low annual mean temperature, the max temperature of the warmest month is 22–33 °C, and the min temperature of the coldest month is − 15 to − 5 °C (Fig. 4). So, *Phelypaea tournefortii* is somewhat resistant to scenarios with low precipitation.

Environmental (i.e. climate, soil, topography) and biological factors (i.e. hosts, competition, mimicry, herbivory) influence the proper development and survival of parasitic plant populations^18,74,75^. Also, different parasite-host pairs vary in the overlap of ecological tolerances influencing their habitat distribution^29^. Temperature and seed moisture content at the beginning of conditioning play key roles in the seed germination of holoparasitic species (e.g. *Orobanche* s.l.)^26^. Moreover, many Orobanchaceae species require cold stratification for germination^76^, so global warming potentially limits its distribution. Holoparasites acquire water mainly from their hosts; therefore, except for seed germination and early seedling growth towards the host, the effects of drought on parasitic plants are probably mainly indirect through the host. Moreover, seeds of *Orobanche* s.l., produced in great numbers, may remain in the soil for decades awaiting the correct host^26^ and suitable climatic conditions.

Drought causes water stress in all plants, but plants’ water demands are higher than others. So, hosts and parasites are susceptible to soil drying^19^. Parasitic plants have characteristically high transpiration rates, and elevated water demands are advantageous, affecting more negative water potentials than their hosts and enabling constant passive water uptake^77^. Holoparasites have lower transpiration rates than hemiparasites because they are underground for much of their lives and, even after emergence, do not photosynthesize due lack of typical leaves (only scales) necessary for effective transpiration^78^. However, prolonged drought becomes a problem, also with sublethal effects, in both parasite and host, due to unregulated and the high water demands of parasitic plants^19,79–81^. Drought and other extreme climatic events can affect parasitic plants^82^. The recent finding also showed that climatic variables rather than photoperiod influence the phenology of some parasitic plants^83^.

In addition, there are already observations related to the influence of climate on the transition of parasitic plants to new hosts, which also changes their ranges^21,84,85^. Highly specialized parasitic plants with narrow host ranges are more sensitive to the effects of climate change^86^. This influence is in arid and semi-arid habitats that will o become progressively more arid^21^. So, microclimatic refuges may play an important role^87,88^. The suitable area coverage of *P. tournefortii* is higher in models based exclusively on climatic data. However, including the *Tanacetum* host in our analyses reduced the available areas due to the habitat loss predicted for the hosts. *P. tournefortii*, based on current observations, shows less significant adaptive potential to other hosts and dispersal opportunities. It shows a strict host specificity to the few *Tanacetum* species that prefer steppe or semi-arid conditions. However, other hosts cited in the literature need confirmation, such as *Anthemis*, *Pyrethrum myriophyllum* [*Tanacetum polycephalum* subsp. *duderanum*]^40^.

Very few studies are available so far about modeling and predicting the impact of climate change on the distribution of holoparasitic plants. Climate change affects these organisms' and their hosts' distribution^32^. Also, not all studies included the host's future potential distribution, and only a few evaluated the influence of global warming on the relationship between parasites and hosts. Different holoparasites species may respond differently to future climate change^29,89^. Liu et al.^31^, in a paper on holoparasitic *Cistanche deserticola*, a root parasitic of *Haloxylon ammodendron,* indicated precipitation-related factors as more important than temperature-related conditions in shaping the distribution of the parasite. Schao et al.^90^ studying future distribution of the same pair of species predicted significant habitat loss (26.6–48.2%) of the niches suitable for the parasite. On the other hand, models of future distribution of *H. ammodendron* in Northwest China presented by Hong et al.^91^ indicated slight expansion of the potential range of this species. As summarized by Lu et al.^29^, climate factors and niche overlaps between them can jointly affect the suitable habitats of parasitic plants and this is certainly true also for *P. tournefortii.*

Mohamed et al.^26^, in a study on the worst global invasive crop species, including *Orobanche cumana*, *O. crenata*, and *O. ramosa*, has shown that with climatic change, *Orobanche* s.l. species could pose a greater negative effect on agriculture by expanding their ranges farther north in Europe and elsewhere^26^. However, the authors did not include the parasite hosts in their analyses. In another research, Zhang et al.^30^ evaluated the potential distribution of invasive weeds (*O*. *cumana* and *Phelipanche aegyptiaca*) in China and their susceptible host plants (*Helianthus annuus* and *Solanum lycopersicon*) using MaxEnt. They found that elevation and topsoil pH were the decisive factors for shaping *O*. *cumana* distribution, and precipitation seasonality and annual precipitation were the dominant bioclimatic variables limiting the spread of *P*. *aegyptiaca*. This study suggested that these two invasive species risk zones will move significantly northward to higher latitudes^30^.

Tsai & Manos^23^ found that host range changes had little effect on the parasite’s *Epifagus virginiana* range expansion and recognized host density as the main driver of parasite spread. Moreover, the authors indicated that the parasite may not follow the host’s migration due to climate change. Renjana et al.^28^ studied the potential distribution of *Rafflesia arnoldii* and its host plants, recognizing the mean temperature, slope, elevation, soil organic carbon content, and soil type as crucial for their occurrence^28^. Mkala et al.^32^used models to analyze the impact of climate change on the distribution of *Hydnora abyssinica*, *H. africana*, and their hosts, indicating that the precipitation of the wettest month was the most important variable contributing to the distribution of this species. According to the author’s observation, the ground moisture is an essential factor affecting their survival chances, which may also apply to many root holoparasites and other relatively soft-tissue plants^32,92^. Lu et al.^29^, in their study on *Boschniakia rossica, C. mongolica, C. deserticola*, and *Cynomorium songaricum* and their hosts, indicated that host plants respond differently to future climate change. This study was the first to evaluate the overlap of niches of holoparasites and their hosts. Holoparasites and their primary hosts respond differently to climate change, which results in an apparent spatial mismatch of their suitable distribution^29^.

Semi-arid steppe has seasonal changes, including cold and dry winters and short warm summers which low rainfall. This variability increases these ecosystems' vulnerability to climate change’s effects. Changes in climate patterns are already evident in the entire South Caucasus countries – Azerbaijan, Armenia, and Georgia. All climate models consistently show that the South Caucasus will become warmer, and annual temperatures are increasing, accompanied by severe heat waves and droughts. Changes in precipitation patterns also relate to climate change, such as heavy rains and unusual hail storms. Armenia harbors most of the *P. tournefortii* populations and is highly vulnerable to global climate change as a mountainous country with an arid climate.

Nevertheless, climate change is already affecting Armenia. The annual temperature is increasing above the global average, with potential warming of 4.7 °C by the 2090s and a significant decrease in precipitation (https://eu4climate.eu/armenia/). There is a general trend towards drier and hotter summers. However, montane regions can expect a slight precipitation increase by the mid-twenty-first century^93^. Regardless, in Azerbaijan, the expected increase is lower, around 3.6 °C. Additionally, in Azerbaijan in 2070–2100, the annual precipitation decrease will be around 15% but 11% in summer. On the other hand, in the winter, the annual precipitation might increase by 22%, the highest value among the montane regions. At the same time, the annual precipitation may rise to 24% in Armenia^94^. The mountainous areas of the South Caucasus have a wide variety of climate zones, from cold alpine to temperate, humid and arid habitats, with contrasting geology or topography^95^. As a result, the Caucasus region is an important reservoir of biodiversity, as a biodiversity hotspot’ based on recorded species richness and endemism level^96^.

Trade-offs between maximizing efficiency at obtaining water from hosts and sensitivity to water stress underlie parasitic plants’ range and host shifts^21^. Many holoparasites possess few species in particular genera and restricted abundance, distribution, and host range^17,18^. So, parasitism in plants is more an evolutionary constraint than a key innovation^21^.

It is difficult to evaluate the possibility of the parasite shifting to new hosts characterized by broader ecological tolerance or to predict potential adaptations related to the biology and ecology of these plants, which could result from climate change. In the flowers and stem of the *P. tournefortii*, there are three pigments classified as anthocyanin, responsible for the very intense red color of the flowers. The large amounts of anthocyanin in the flowers of *P. tournefortii* seem unique^41^. Anthocyanins contribute to stress tolerance in plants, possibly also to pressures related to global warming. At high altitudes, flavonoids improve photoprotection and protect plants against lower temperatures^97^. Phenylethanoid glycosides (PhGs) may be specialized metabolites synthesized by parasitic species, and they usually do not occur in the host tissue^98^. These metabolites perform a protective, antimicrobial, anti-stress, or stress-resistant function^99^.

Moreover, increased accumulation of organic and inorganic elements in parasites, with condensation of many cations, greatly exceeding concentrations in host tissues. Additionally, producing specific metabolites (PhGs) by parasites may have an enhanced protective effect against drought, pests, herbivores, and diseases. This accumulation of organic and inorganic elements and the production of specific metabolites affect the parasite-host relationship and the entire ecosystem^21,100^. Furthermore, the knowledge of the diversity and role of bacterial seed endophytes of holoparasitic plant species is still limited^101–104^. These bacteria' plant growth-promoting (PGP) traits can improve plant tolerance against abiotic stresses. However, their benefits for seed germination and seedling development require further research^103^.

While *P. tournefortii* occurs in the Southern Caucasus and Turkey and is a parasite of *Tanacetum* L.^24,105^, the other two representatives of the genus show different geographical distributions. *P. coccinea* occurs in the Caucasus and Crimea and is a parasite of *Psephellus* Cass. and *Centaurea* L., rarely *Klasea* Cass. *P. boissieri* occurs in the Balkans (Albania, Greece, North Macedonia) and Western Asia (Turkey, Iraq, and Iran) and parasitizes *Centaurea* and occasionally *Cousinia* Cass. in Iraq^24,105^. However, *P. boissieri* is molecularly, morphologically and regarding host preferences similar to *P. coccinea*^24,25^. Therefore, further research into the variability of *P. boissieri* is required^25,106^. Of all *Phelypaea* species, *P. coccinea* shows the northernmost distribution, except for semi-arid habitats, also preferring colder climates, more mesophilic, and higher elevation habitats. In the future, we recommend using the ecological niche modeling (ENM) approach to estimate the effects of climate change on the suitability of all species from the genus *Phelypaea. *Also, to identify the key climatic factors affecting the suitable habitat of this parasitic species and their primary hosts.

## Conclusions

Predicting how climate change will alter the relationships between hosts and holoparasitic plants is complex since the effects can generate different responses in the host, parasite and, symbiosis. Additionally, it is hard to predict potential adaptation at different levels (i.e. molecular, microbiological, phytochemical, or ecological). Thus, we present a simulation of changes in the suitable areas of *P. tournefortii* using climate data and an ecological factor (hosts). Based on our models, *P. tournefortii* and its hosts will lose significantly suitable areas due to global warming. These losses are higher in central and southern Armenia, which has most of the population, in Nakhchivan in Azerbaijan, northern Iran, and north-eastern Turkey. However, the scenarios were less catastrophic when we included the host distribution as a variable in the models. In some scenarios, *P. tournefortii* distribution showed more expansion and less retraction. So, to model parasitic species, it is essential to include host distribution as a variable. *P. tournefortii*, based on current observations, does not show potential adaptation to other hosts and additional dispersal opportunities. It is a strict host species, particularly *Tanacetum* species. It prefers steppe and semi-desert habitats with relatively narrow climatic conditions. So, *P. tournefortii* is sensitive to climate change. In this context, it is necessary to enhance the conservation of its primary hosts and their preferred habitats. However, a change in the potential adaptation for new hosts, more resistant to climate change or with a broader range of habitat preferences, cannot be ruled out under the influence of climatic or other factors. In the future, performing similar analyses on other *Phelypaea* species may allow us to draw broader conclusions about the regularities in the specificity of shaping its range and the impact of host availability in a changing climate.

## Supplementary Information

Supplementary Information 1.

Supplementary Information 2.

Supplementary Information 3.

Supplementary Information 4.

Supplementary Information 5.

Supplementary Information 6.

## Data Availability

All relevant data are presented in the manuscript and supplementary files.
