# Supplementary material for: Effect of global warming on the potential distribution of a holoparasitic plant (Phelypaea tournefortii): both climate and host distribution matter
Source: Sci Rep. 2023 Jul 3;13:10741. doi: 10.1038/s41598-023-37897-1 (PMC10318063; doi:10.1038/s41598-023-37897-1)
Supplement: Supplementary file 1 — Supplementary Information 1. [file 41598_2023_37897_MOESM1_ESM.docx]

**Effect of global warming on the potential distribution of a holoparasitic plant *Phelypaea tournefortii* – both climate and host distribution matter**

**Renata Piwowarczyk^1^ & Marta Kolanowska**^2*^

^1^ Center for Research and Conservation of Biodiversity, Department of Environmental Biology, Institute of Biology, Jan Kochanowski University, Uniwersytecka 7 Street, PL-25-406, Kielce, Poland

^2^ University of Lodz, Faculty of Biology and Environmental Protection, Department of Geobotany and Plant Ecology, Banacha 12/16, PL-90-237 Lodz, Poland

***** email: [martakolanowska@wp.pl](mailto:martakolanowska@wp.pl)

**S1 Annex.** Localities of species studied used in modeling.

| **Species** | **Decimal Longitude** | | **Decimal Latitude** |
| --- | --- | --- | --- |
| *Phelypaea tournefortii* | | 44.24027777777778 | 40.3811111 |
| *Phelypaea tournefortii* | | 44.904019444444444 | 39.9214444 |
| *Phelypaea tournefortii* | | 45.061388888888885 | 39.8622222 |
| *Phelypaea tournefortii* | | 45.0375 | 39.8366667 |
| *Phelypaea tournefortii* | | 45.00416666666667 | 39.9755556 |
| *Phelypaea tournefortii* | | 44.94638888888888 | 39.7683333 |
| *Phelypaea tournefortii* | | 44.97694444444445 | 39.8038889 |
| *Phelypaea tournefortii* | | 44.80555555555555 | 40.1002778 |
| *Phelypaea tournefortii* | | 44.91694444444444 | 39.8572222 |
| *Phelypaea tournefortii* | | 44.77472222222222 | 40.005 |
| *Phelypaea tournefortii* | | 44.87277777777778 | 40.0344444 |
| *Phelypaea tournefortii* | | 44.80611111111111 | 40.1372222 |
| *Phelypaea tournefortii* | | 44.74277777777778 | 40.115 |
| *Phelypaea tournefortii* | | 44.76416666666667 | 40.1202778 |
| *Phelypaea tournefortii* | | 44.76888888888889 | 40.1225 |
| *Phelypaea tournefortii* | | 44.676944444444445 | 40.4755556 |
| *Phelypaea tournefortii* | | 44.70583333333334 | 40.4066667 |
| *Phelypaea tournefortii* | | 44.565916666666666 | 40.515 |
| *Phelypaea tournefortii* | | 44.58972222222222 | 40.2936111 |
| *Phelypaea tournefortii* | | 44.71222222222222 | 40.5313889 |
| *Phelypaea tournefortii* | | 44.44583333333333 | 40.3844444 |
| *Phelypaea tournefortii* | | 44.456944444444446 | 40.4027778 |
| *Phelypaea tournefortii* | | 45.405 | 39.78 |
| *Phelypaea tournefortii* | | 45.375 | 39.6575 |
| *Phelypaea tournefortii* | | 45.63722222222222 | 39.8225 |
| *Phelypaea tournefortii* | | 45.35777777777778 | 39.8902778 |
| *Phelypaea tournefortii* | | 45.361111111111114 | 39.8086111 |
| *Phelypaea tournefortii* | | 45.32138888888889 | 39.6352778 |
| *Phelypaea tournefortii* | | 45.09905555555556 | 39.8012778 |
| *Phelypaea tournefortii* | | 45.48563888888889 | 39.8420278 |
| *Phelypaea tournefortii* | | 45.20655555555556 | 39.6540556 |
| *Phelypaea tournefortii* | | 45.48441666666667 | 39.5728056 |
| *Phelypaea tournefortii* | | 45.48788888888889 | 39.6105 |
| *Phelypaea tournefortii* | | 45.306916666666666 | 39.67375 |
| *Phelypaea tournefortii* | | 45.505833333333335 | 39.9205556 |
| *Phelypaea tournefortii* | | 45.46805555555556 | 39.8838889 |
| *Phelypaea tournefortii* | | 45.45888888888889 | 39.5830556 |
| *Phelypaea tournefortii* | | 45.086666666666666 | 39.775 |
| *Phelypaea tournefortii* | | 44.55416666666667 | 40.2108333 |
| *Phelypaea tournefortii* | | 44.53638888888889 | 40.2305556 |
| *Phelypaea tournefortii* | | 45.016333 | 39.975972 |
| *Phelypaea tournefortii* | | 44.722201 | 40.524312 |
| *Phelypaea tournefortii* | | 44.65416666666667 | 40.1697222 |
| *Phelypaea tournefortii* | | 45.374154 | 39.831684 |
| *Phelypaea tournefortii* | | 45.233333333333334 | 39.9358333 |
| *Phelypaea tournefortii* | | 45.364444444444445 | 39.83 |
| *Phelypaea tournefortii* | | 44.63084166666667 | 40.1765111 |
| *Phelypaea tournefortii* | | 45.39277777777778 | 39.5022222 |
| *Phelypaea tournefortii* | | 45.403055555555554 | 39.3419444 |
| *Phelypaea tournefortii* | | 45.2592 | 39.5114 |
| *Phelypaea tournefortii* | | 40.24888888888889 | 40.2547222 |
| *Phelypaea tournefortii* | | 40.42611111111111 | 40.0802778 |
| *Phelypaea tournefortii* | | 42.19138888888889 | 39.3880556 |
| *Phelypaea tournefortii* | | 40.44 | 40.0586111 |
| *Phelypaea tournefortii* | | 42.483333333333334 | 40.3338889 |
| *Phelypaea tournefortii* | | 43.79388888888889 | 38.9875 |
| *Phelypaea tournefortii* | | 45.28916666666667 | 39.4727778 |
| *Phelypaea tournefortii* | | 45.08416666666667 | 39.4897222 |
| *Phelypaea tournefortii* | | 44.72943 | 40.541237 |
| *Phelypaea tournefortii* | | 44.720771 | 40.523494 |
| *Phelypaea tournefortii* | | 45.56083333333333 | 39.4144444 |
| *Phelypaea tournefortii* | | 43.16222222222222 | 40.1422222 |
| *Phelypaea tournefortii* | | 44.93722222222222 | 39.9611111 |
| *Tanacetum argyrophyllum* | | 48.202987 | 38.7582 |
| *Tanacetum argyrophyllum* | | 46.21528 | 38.902067 |
| *Tanacetum argyrophyllum* | | 46.013001 | 39.030898 |
| *Tanacetum argyrophyllum* | | 45.891867 | 39.076076 |
| *Tanacetum argyrophyllum* | | 45.94972 | 39.50167 |
| *Tanacetum argyrophyllum* | | 45.1059 | 39.6227 |
| *Tanacetum argyrophyllum* | | 45.1059 | 39.6227 |
| *Tanacetum argyrophyllum* | | 45.2325 | 39.68472 |
| *Tanacetum argyrophyllum* | | 45.567975 | 39.698023 |
| *Tanacetum argyrophyllum* | | 45.625 | 39.71722 |
| *Tanacetum argyrophyllum* | | 45.36667 | 39.765 |
| *Tanacetum argyrophyllum* | | 44.96667 | 39.8 |
| *Tanacetum argyrophyllum* | | 45.413401 | 39.833577 |
| *Tanacetum argyrophyllum* | | 45.413401 | 39.833577 |
| *Tanacetum argyrophyllum* | | 44.833832 | 39.837805 |
| *Tanacetum argyrophyllum* | | 44.96528 | 39.85972 |
| *Tanacetum argyrophyllum* | | 45.246334 | 39.90358 |
| *Tanacetum argyrophyllum* | | 44.88333 | 39.95 |
| *Tanacetum argyrophyllum* | | 44.88 | 39.95 |
| *Tanacetum argyrophyllum* | | 44.61556 | 40.03889 |
| *Tanacetum argyrophyllum* | | 44.734955 | 40.111951 |
| *Tanacetum argyrophyllum* | | 44.7 | 40.11667 |
| *Tanacetum argyrophyllum* | | 44.7 | 40.12 |
| *Tanacetum argyrophyllum* | | 44.15361 | 40.30944 |
| *Tanacetum argyrophyllum* | | 44.1775 | 40.33306 |
| *Tanacetum argyrophyllum* | | 43.78194 | 40.34889 |
| *Tanacetum argyrophyllum* | | 44.06139 | 40.36139 |
| *Tanacetum argyrophyllum* | | 44.06139 | 40.36139 |
| *Tanacetum argyrophyllum* | | 44.05889 | 40.36917 |
| *Tanacetum argyrophyllum* | | 43.78 | 40.38 |
| *Tanacetum argyrophyllum* | | 43.78333 | 40.38333 |
| *Tanacetum argyrophyllum* | | 44.227696 | 40.38722 |
| *Tanacetum argyrophyllum* | | 44.22611 | 40.38889 |
| *Tanacetum argyrophyllum* | | 44.6 | 40.48333 |
| *Tanacetum argyrophyllum* | | 45.271912 | 40.50127 |
| *Tanacetum argyrophyllum* | | 45.271912 | 40.50127 |
| *Tanacetum argyrophyllum* | | 44.717274 | 40.531937 |
| *Tanacetum argyrophyllum* | | 44.97 | 40.58 |
| *Tanacetum argyrophyllum* | | 43.3175 | 41.39222 |
| *Tanacetum argyrophyllum* | | 43.10028 | 41.61417 |
| *Tanacetum chiliophyllum* | | 46.34005366189273 | 39.101889939482675 |
| *Tanacetum chiliophyllum* | | 45.98444 | 39.36528 |
| *Tanacetum chiliophyllum* | | 45.90268139771593 | 39.40602398823518 |
| *Tanacetum chiliophyllum* | | 45.87278 | 39.40889 |
| *Tanacetum chiliophyllum* | | 45.25080133613872 | 39.66435631115773 |
| *Tanacetum chiliophyllum* | | 45.28333 | 39.66667 |
| *Tanacetum chiliophyllum* | | 45.30139 | 39.69194 |
| *Tanacetum chiliophyllum* | | 45.68667 | 39.70444 |
| *Tanacetum chiliophyllum* | | 45.33705696060192 | 39.762800600962166 |
| *Tanacetum chiliophyllum* | | 39.82905516667172 | 44.97922339549816 |
| *Tanacetum chiliophyllum* | | 41.27568865004911 | 39.844883517798074 |
| *Tanacetum chiliophyllum* | | 45.35535370903274 | 39.88933867662239 |
| *Tanacetum chiliophyllum* | | 44.87349870520627 | 39.94643709074473 |
| *Tanacetum chiliophyllum* | | 44.28333 | 40.38333 |
| *Tanacetum chiliophyllum* | | 44.67361 | 40.46472 |
| *Tanacetum chiliophyllum* | | 44.983796241415476 | 40.57742159675165 |
| *Tanacetum chiliophyllum* | | 44.950001 | 40.580002 |
| *Tanacetum chiliophyllum* | | 44.562840369928495 | 40.641028278215344 |
| *Tanacetum chiliophyllum* | | 44.0 | 40.883333 |
| *Tanacetum chiliophyllum* | | 43.38667 | 41.60639 |
| *Tanacetum chiliophyllum* | | 57.647458918146924 | 42.334683952874144 |
