# Supplementary material for: Effect of global warming on the potential distribution of a holoparasitic plant (Phelypaea tournefortii): both climate and host distribution matter
Source: Sci Rep. 2023 Jul 3;13:10741. doi: 10.1038/s41598-023-37897-1 (PMC10318063; doi:10.1038/s41598-023-37897-1)
Supplement: Supplementary file 2 — Supplementary Information 2. [file 41598_2023_37897_MOESM2_ESM.docx]

**Effect of global warming on the potential distribution of a holoparasitic plant *Phelypaea tournefortii* – both climate and host distribution matter**

**Renata Piwowarczyk^1^ & Marta Kolanowska**^2*^

^1^ Center for Research and Conservation of Biodiversity, Department of Environmental Biology, Institute of Biology, Jan Kochanowski University, Uniwersytecka 7 Street, PL-25-406, Kielce, Poland

^2^ University of Lodz, Faculty of Biology and Environmental Protection, Department of Geobotany and Plant Ecology, Banacha 12/16, PL-90-237 Lodz, Poland

***** email: [martakolanowska@wp.pl](mailto:martakolanowska@wp.pl)

**S2 Annex.** Bioclimatic variables. Layers used in ENM analyses marked with ‘+”.

| **Code** | **Description** | **ENM** |
| --- | --- | --- |
| bio1 | annual mean temperature | + |
| bio2 | mean diurnal range [mean of monthly (max temp - min temp)] | + |
| bio3 | isothermality (bio2/bio7) (×100) |  |
| bio4 | temperature seasonality (standard deviation ×100) | + |
| bio5 | max temperature of warmest month |  |
| bio6 | min temperature of coldest month |  |
| bio7 | temperature annual range (bio5-bio6) |  |
| bio8 | mean temperature of wettest quarter | + |
| bio9 | mean temperature of driest quarter | + |
| bio10 | mean temperature of warmest quarter |  |
| bio11 | mean temperature of coldest quarter |  |
| bio12 | annual precipitation | + |
| bio13 | precipitation of wettest month |  |
| bio14 | precipitation of driest month |  |
| bio15 | precipitation seasonality (coefficient of variation) | + |
| bio16 | precipitation of wettest quarter |  |
| bio17 | precipitation of driest quarter |  |
| bio18 | precipitation of warmest quarter |  |
| bio19 | precipitation of coldest quarter |  |
