# Supplementary material for: Effect of global warming on the potential distribution of a holoparasitic plant (Phelypaea tournefortii): both climate and host distribution matter
Source: Sci Rep. 2023 Jul 3;13:10741. doi: 10.1038/s41598-023-37897-1 (PMC10318063; doi:10.1038/s41598-023-37897-1)
Supplement: Supplementary file 3 — Supplementary Information 3. [file 41598_2023_37897_MOESM3_ESM.docx]

**Effect of global warming on the potential distribution of a holoparasitic plant *Phelypaea tournefortii* – both climate and host distribution matter**

**Renata Piwowarczyk^1^ & Marta Kolanowska**^2*^

^1^ Center for Research and Conservation of Biodiversity, Department of Environmental Biology, Institute of Biology, Jan Kochanowski University, Uniwersytecka 7 Street, PL-25-406, Kielce, Poland

^2^ University of Lodz, Faculty of Biology and Environmental Protection, Department of Geobotany and Plant Ecology, Banacha 12/16, PL-90-237 Lodz, Poland

***** email: [martakolanowska@wp.pl](mailto:martakolanowska@wp.pl)

**S3 Annex.** Results of Pearson's Correlation Coefficient (R) statistics.

|  | bio1 | bio2 | bio3 | bio4 | bio5 | bio6 | bio7 | bio8 | bio9 | bio10 | bio11 | bio12 | bio13 | bio14 | bio15 | bio16 | bio17 | bio18 | bio19 |
| --- | --- | --- | --- | --- | --- | --- | --- | --- | --- | --- | --- | --- | --- | --- | --- | --- | --- | --- | --- |
| bio1 | x | 0,080 | 0,119 | -0,085 | 0,775 | 0,852 | -0,083 | -0,018 | 0,655 | 0,886 | 0,893 | -0,223 | -0,081 | -0,419 | 0,512 | -0,079 | -0,413 | -0,456 | 0,148 |
| bio2 | x | x | 0,816 | 0,292 | 0,505 | -0,250 | 0,663 | -0,284 | 0,210 | 0,193 | -0,087 | -0,006 | 0,061 | -0,218 | 0,484 | 0,073 | -0,227 | -0,218 | 0,190 |
| bio3 | x | x | x | -0,286 | 0,204 | 0,061 | 0,123 | -0,414 | 0,214 | -0,041 | 0,206 | 0,376 | 0,424 | 0,077 | 0,570 | 0,435 | 0,082 | 0,097 | 0,463 |
| bio4 | x | x | x | x | 0,477 | -0,546 | 0,902 | 0,309 | -0,086 | 0,385 | -0,522 | -0,577 | -0,586 | -0,365 | -0,264 | -0,581 | -0,386 | -0,401 | -0,438 |
| bio5 | x | x | x | x | x | 0,355 | 0,555 | 0,021 | 0,562 | 0,936 | 0,443 | -0,444 | -0,307 | -0,593 | 0,457 | -0,300 | -0,600 | -0,644 | -0,010 |
| bio6 | x | x | x | x | x | x | -0,581 | -0,076 | 0,540 | 0,541 | 0,981 | 0,063 | 0,168 | -0,147 | 0,440 | 0,166 | -0,132 | -0,163 | 0,280 |
| bio7 | x | x | x | x | x | x | x | 0,086 | 0,008 | 0,333 | -0,488 | -0,443 | -0,418 | -0,386 | 0,006 | -0,409 | -0,406 | -0,415 | -0,258 |
| bio8 | x | x | x | x | x | x | x | x | -0,467 | 0,133 | -0,147 | -0,245 | -0,301 | 0,112 | -0,542 | -0,319 | 0,117 | 0,242 | -0,448 |
| bio9 | x | x | x | x | x | x | x | x | x | 0,566 | 0,597 | -0,177 | -0,033 | -0,487 | 0,627 | -0,032 | -0,473 | -0,597 | 0,202 |
| bio10 | x | x | x | x | x | x | x | x | x | x | 0,585 | -0,479 | -0,353 | -0,557 | 0,345 | -0,349 | -0,561 | -0,607 | -0,075 |
| bio11 | x | x | x | x | x | x | x | x | x | x | x | 0,063 | 0,187 | -0,194 | 0,545 | 0,187 | -0,180 | -0,211 | 0,317 |
| bio12 | x | x | x | x | x | x | x | x | x | x | x | x | 0,935 | 0,742 | 0,080 | 0,947 | 0,756 | 0,725 | 0,829 |
| bio13 | x | x | x | x | x | x | x | x | x | x | x | x | x | 0,512 | 0,336 | 0,995 | 0,528 | 0,555 | 0,850 |
| bio14 | x | x | x | x | x | x | x | x | x | x | x | x | x | x | -0,491 | 0,522 | 0,995 | 0,894 | 0,384 |
| bio15 | x | x | x | x | x | x | x | x | x | x | x | x | x | x | x | 0,337 | -0,489 | -0,401 | 0,403 |
| bio16 | x | x | x | x | x | x | x | x | x | x | x | x | x | x | x | x | 0,536 | 0,558 | 0,875 |
| bio17 | x | x | x | x | x | x | x | x | x | x | x | x | x | x | x | x | x | 0,897 | 0,394 |
| bio18 | x | x | x | x | x | x | x | x | x | x | x | x | x | x | x | x | x | x | 0,276 |
| bio19 | x | x | x | x | x | x | x | x | x | x | x | x | x | x | x | x | x | x | x |
