# Supplementary material for: Effect of global warming on the potential distribution of a holoparasitic plant (Phelypaea tournefortii): both climate and host distribution matter
Source: Sci Rep. 2023 Jul 3;13:10741. doi: 10.1038/s41598-023-37897-1 (PMC10318063; doi:10.1038/s41598-023-37897-1)
Supplement: Supplementary file 4 — Supplementary Information 4. [file 41598_2023_37897_MOESM4_ESM.docx]

**Effect of global warming on the potential distribution of a holoparasitic plant *Phelypaea tournefortii* – both climate and host distribution matter**

**Renata Piwowarczyk^1^ & Marta Kolanowska**^2*^

^1^ Center for Research and Conservation of Biodiversity, Department of Environmental Biology, Institute of Biology, Jan Kochanowski University, Uniwersytecka 7 Street, PL-25-406, Kielce, Poland

^2^ University of Lodz, Faculty of Biology and Environmental Protection, Department of Geobotany and Plant Ecology, Banacha 12/16, PL-90-237 Lodz, Poland

***** email: [martakolanowska@wp.pl](mailto:martakolanowska@wp.pl)

**S4 Annex.** Comparison of analysed climate change simulations. Legend: pr = precipitation, tasmax = max temperature. Maps downloaded from WorldClim 2.

**
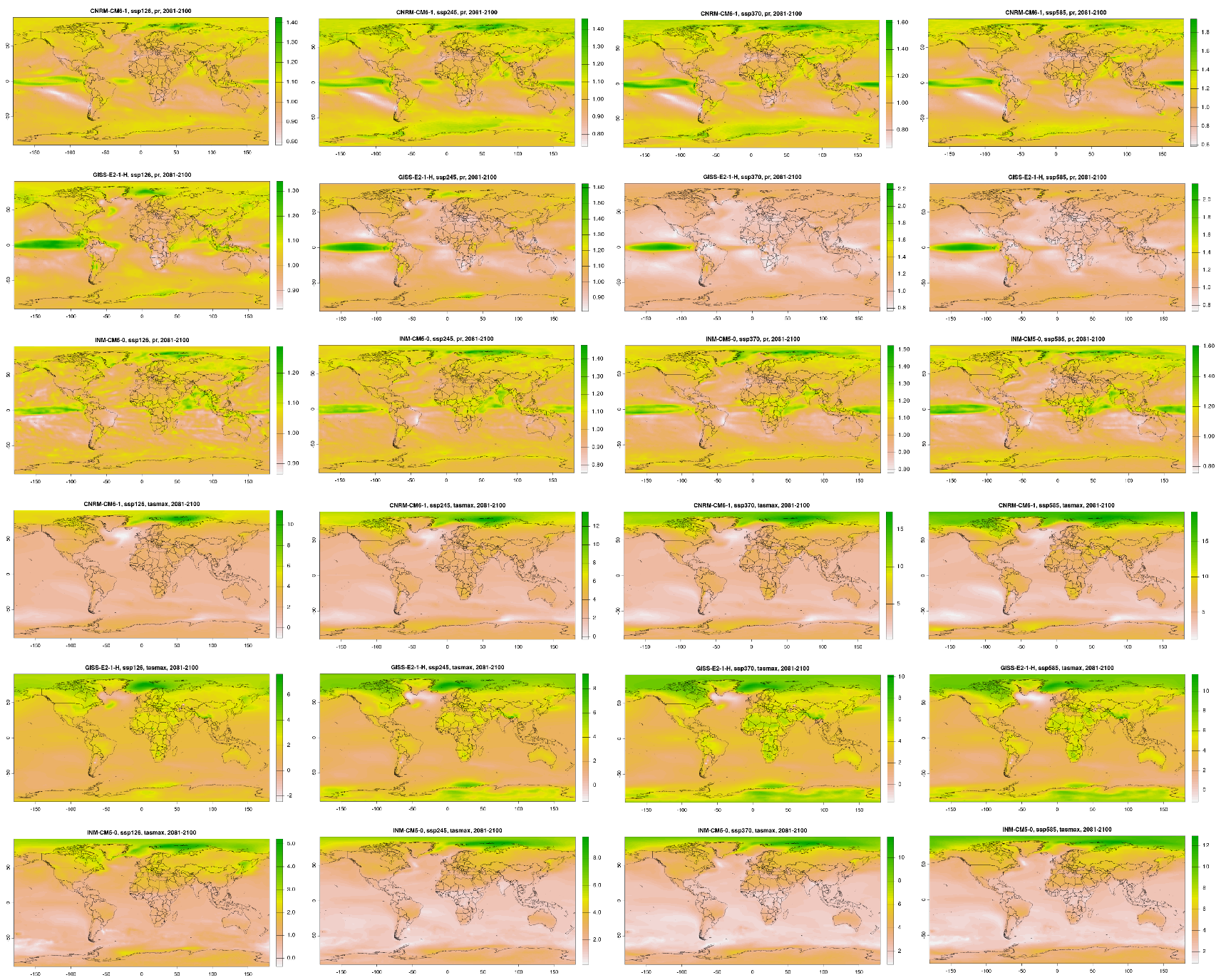
**
