# Supplementary material for: Effect of global warming on the potential distribution of a holoparasitic plant (Phelypaea tournefortii): both climate and host distribution matter
Source: Sci Rep. 2023 Jul 3;13:10741. doi: 10.1038/s41598-023-37897-1 (PMC10318063; doi:10.1038/s41598-023-37897-1)
Supplement: Supplementary file 6 — Supplementary Information 6. [file 41598_2023_37897_MOESM6_ESM.docx]

**Effect of global warming on the potential distribution of a holoparasitic plant *Phelypaea tournefortii* – both climate and host distribution matter**

**Renata Piwowarczyk^1^ & Marta Kolanowska**^2*^

^1^ Center for Research and Conservation of Biodiversity, Department of Environmental Biology, Institute of Biology, Jan Kochanowski University, Uniwersytecka 7 Street, PL-25-406, Kielce, Poland

^2^ University of Lodz, Faculty of Biology and Environmental Protection, Department of Geobotany and Plant Ecology, Banacha 12/16, PL-90-237 Lodz, Poland

***** email: [martakolanowska@wp.pl](mailto:martakolanowska@wp.pl)

**S6 Annex.** Changes in the coverage (km^2^) of suitable niches for *Phelypaea tournefortii* hosts*.*

| **Model** | **Projection** | **Scenario** | **Range expansion** | **Range contraction** | **Overall change** |
| --- | --- | --- | --- | --- | --- |
| *Tanacetum argyrophyllum* | CNRM | SSP1-2.6 | 1291.938 | 12884.237 | -46% |
|  |  | SSP2-4.5 | 734.191 | 19631.770 | -75% |
|  |  | SSP3-7.0 | 38.878 | 25005.125 | -98% |
|  |  | SSP5-8.5 | 0.000 | 25349.791 | -100% |
| *Tanacetum chiliophyllum* | CNRM | SSP1-2.6 | 6592.770 | 27034.992 | -36% |
|  |  | SSP2-4.5 | 3738.993 | 37817.587 | -60% |
|  |  | SSP3-7.0 | 4091.884 | 45083.989 | -72% |
|  |  | SSP5-8.5 | 316.256 | 56277.044 | -99% |
| *Tanacetum argyrophyllum* | GISS-E2 | SSP1-2.6 | 938.300 | 16094.642 | -60% |
|  |  | SSP2-4.5 | 1682.958 | 16562.671 | -59% |
|  |  | SSP3-7.0 | 1641.837 | 19315.515 | -70% |
|  |  | SSP5-8.5 | 2103.137 | 20295.683 | -72% |
| *Tanacetum chiliophyllum* | GISS-E2 | SSP1-2.6 | 2885.178 | 30678.286 | -49% |
|  |  | SSP2-4.5 | 4406.644 | 35348.850 | -55% |
|  |  | SSP3-7.0 | 3984.222 | 38969.715 | -62% |
|  |  | SSP5-8.5 | 8796.092 | 42550.206 | -60% |
| *Tanacetum argyrophyllum* | INM | SSP1-2.6 | 525.597 | 15659.511 | -60% |
|  |  | SSP2-4.5 | 1943.140 | 18368.991 | -65% |
|  |  | SSP3-7.0 | 2334.161 | 18068.436 | -62% |
|  |  | SSP5-8.5 | 1115.492 | 24030.938 | -90% |
| *Tanacetum chiliophyllum* | INM | SSP1-2.6 | 956.243 | 31159.024 | -53% |
|  |  | SSP2-4.5 | 1835.479 | 43692.614 | -74% |
|  |  | SSP3-7.0 | 4622.714 | 41941.620 | -66% |
|  |  | SSP5-8.5 | 2331.918 | 49721.656 | -84% |
